# The Impact of Coronal Configuration of the Proximal Femur on its Mechanical Properties and the Validation of a New Theoretical Model: Finite Element Analysis and Biomechanical Examination

**DOI:** 10.1111/os.13537

**Published:** 2022-10-17

**Authors:** Lijia Zhang, Baozhang Zhu, Liwan Chen, Wenqing Wang, Xiaoyong Zhang, Jianguo Zhang

**Affiliations:** ^1^ 4+4 Medical Doctor Program Chinese Academy of Medical Science & Peking Union Medical College Beijing China; ^2^ Department of Orthopaedics Peiking Union Medical College Hospital Beijing China; ^3^ Beijing Naton Medical Institute Co., Ltd, Haidian District Beijing China

**Keywords:** Biomechanics, Finite element analysis, Hip fracture, New hypothesis

## Abstract

**Objective:**

This study aims to establish the coronal configuration of the proximal femur as an independent factor for its mechanical properties and provide validation for the theoretical model “fulcrum‐balance‐reconstruction.”

**Methods:**

The digital 3D femur model constructed with the lower extremity high‐resolution computed tomography of a senior subject was applied with the axial compression of 2100N under 5 different *α* angles of 10°, 5°, 0°, −5°, −10°. The equivalent stress distribution of the femoral geometric model under each angle were calculated. Under the same five *α* angles, fatigue test was performed on 15 composite artificial left femurs (three specimens in each angle group) to obtain the failure cycle and fracture site. The statistical analysis was accomplished using One‐Way ANOVA.

**Results:**

The maximum stress of the entire femur in physiological angle (*α* = 10°) occurred below femoral neck with a value of 63.91 MPa. When the proximal femur is in extreme abducted angle (*α* = −10°), the maximum stress shift to the lower medial cortex of femoral shaft with a value of 105.2 MPa. As the *α* angle changed from 10° to −10°, the greater trochanteric region had the largest increment in maximum stress (2.78 times for cortex and 1.67 times for cancellous bone) locally at the proximal femur. The failure cycles of the artificial femurs with a variety of abduction angle were averagely 9126 ± 2453.87 (*α* = −10°), 58,112.33 ± 1293.84 (*α* = −5°), 92,879.67 ± 2398.54 (*α* = 0°), 172,045.3 ± 11011.11 (*α* = 5°), and 264,949.3 ± 35,067.26 (*α* = 10°), and the statistical analysis revealed that the *α* angle of the group of concern is proportional to the P value of the corresponding group compared to the 10° group(*α* = 5° & *α* = 10°, *P* = 0.01; *α* = 0 & *α* = 10°, *P* = 0.001; *α* = −5°, −10° & *α* = 10°, *P* < 0.001). In fatigue test, the fracture appeared on femoral neck for the *α* angles of 10° (three subcapital), 5° (two basal; one transcervical), and 0° (one transcervical). Fracture sites located at trochanteric region were observed with the more abducted angles including 0° (two subtrochanteric) and −5° (two intertrochanteric; one subtrochanteric). The fracture line was only found on femoral shaft in the −10° group.

**Conclusion:**

With increasing hip abduction, the proximal femur shows declining mechanical properties, which suggests higher risk of hip fracture and increasement in the fraction of trochanteric fracture subtype. Furthermore, the hypothesis of “fulcrum‐balance‐reconstruction” was validated by our study to a certain extent.

## Graphical abstract

By exploring the impact of coronal configuration of the proximal femur on its mechanical properties, we provide validation for a new theoretical model and insight of the correlation between lower body force line and hip fracture occurrence.

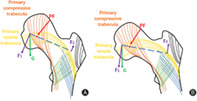

## Introduction

Proximal femoral fracture is an important disease that critically affects the life expectancy and living quality of the elderly. Previous studies suggested that 6% of elderly men and 18% of elderly women are suffered from proximal femoral fractures. With the aging of the world's population, it is estimated that the number of patients with proximal femoral fractures would reach 6.3 million by 2050.^1^ Such tremendous amounts of proximal femoral fracture will bring a heavy burden to society. In order to formulate targeted preventive measures to reduce its occurrence, it is extremely necessary to study the factors that affect the biomechanical properties of the proximal femur.

The strength of the proximal femur is the most valuable mechanical factor for predicting the occurrence of proximal femoral fracture. According to a previous study, the proximal femur is one of the strongest weight‐bearing bone tissue in body. During regular walking, the proximal femur bears a load equivalent to 2–3 times of body weight (BW).^2^ Another *in vivo* experiment shows that the proximal femur is subjected to a load of 8.7 BW during activities.^3^ Moreover, a biomechanical experimental study using cadaveric bone found that the failure load of the proximal femur even exceeds 12 kN.^4^ Many anatomical indications have been identified as the factors affecting the strength of the proximal femur, in which the bone mineral density of femoral neck is found to be significantly related to the strength of the proximal femur,^5^, ^6^, ^7^, ^8^ including the BMD measured by DXA and the densitometric variables measured by QCT.^9^ In addition, the geometric variables measured by QCT,^9^ the volumetric BMD (vBMD), cross‐sectional area, cortical thickness and large cortical bone hole of tibia,^10^ the femoral neck cortical thickness and trabecular bone volume fraction,^11^ the total cortical porosity,^12^ the diameter of femoral neck,^13^ the trochanteric cortical area, femoral neck width,^14^ the femoral neck length, femoral head diameter,^15^ and the cortical thickness,^16^ etc., are also considered as the influencing factors of the strength of the proximal femur. Besides BMD, the other factors mentioned above are very difficult to be altered via clinical intervention. The anatomical evidence orientated research might deduce its relevance with the strength of the proximal femur. However, this has limited significance in guiding clinical practice.

**Fig. 1:**
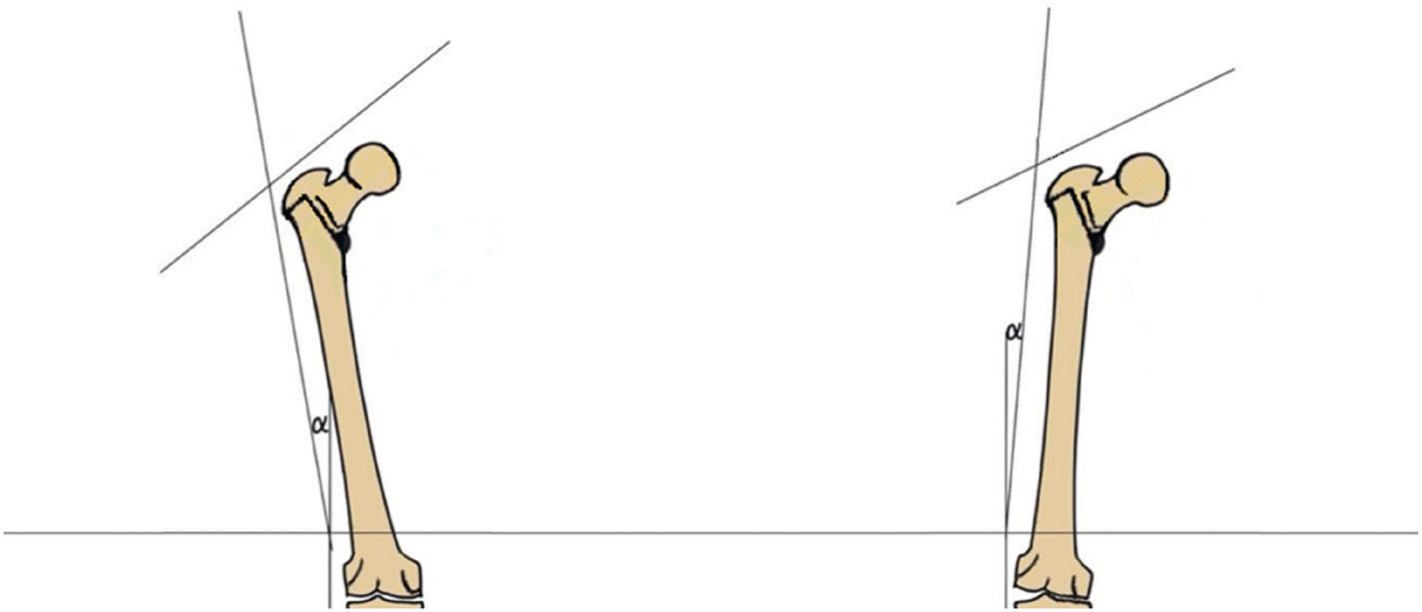
Femur in a coronal configuration near physiological state (left); femur in a coronal configuration of passive abduction (right).

In contrast to the factors mentioned above, the configuration of the proximal femur can be altered easily via surgical approach as knee osteoarthritis with severe genu varum or valgum leads to compensatory hip abduction or adduction.^17^ Furthermore, the high incidence of radiographic osteoarthritis of the knee in the general population^18^ suggests the prevalence of pathological configuration of proximal femurs. Therefore, establishing the configuration of the proximal femur as an independent factor that potentially influences its biomechanical property is of great clinical significance for the prevention of hip fracture. Recent studies have explored the influences that the configuration of the proximal femur has on its mechanical property. Mirzaei *et al*. analyzed the change in strength of the proximal femur in different configurations using finite element analysis (FEA). They found that the proximal femur has the greatest strength with a coronal angle of 20° and a sagittal angle of 10°.^19^ Levadnyi *et al*. used biomechanics experiment and FEA to explore the change in stiffness and surface strain of the proximal femur under different configurational conditions. It was found that the proximal femur has the greatest compressive stiffness when the coronal angle is about 10°.^20^ On the one hand, the main purpose of these studies is only to verify the validity of the finite element models. On the other hand, a reasonable mechanism that explains the effects of configuration of the proximal femur on its mechanical properties was not provided. In this study, our objectives include: (i) exploring the impact of coronal configuration of the proximal femur on its mechanical properties and the incidence rate/subtype of hip fracture; and (ii) validating the “fulcrum‐balance‐reconstruction” model with part of this impact. To quantify the spatial configuration of the proximal femur within the coronal plane, we defined the angle between the femoral shaft and the vertical axis in the coronal plane as angle *α*. Under physiological condition, this angle is approximately 9–10° (Fig. 1 left). During hip abduction, the *α* angle would gradually decrease (Fig. 1 right). By applying FEA and the biomechanical experiment, we evaluated a series of mechanical indicators including stress distribution, failure cycle and fracture site of femur under different *α* angles.

## Materials and Methods

### Finite Element Analysis

**Fig. 2:**
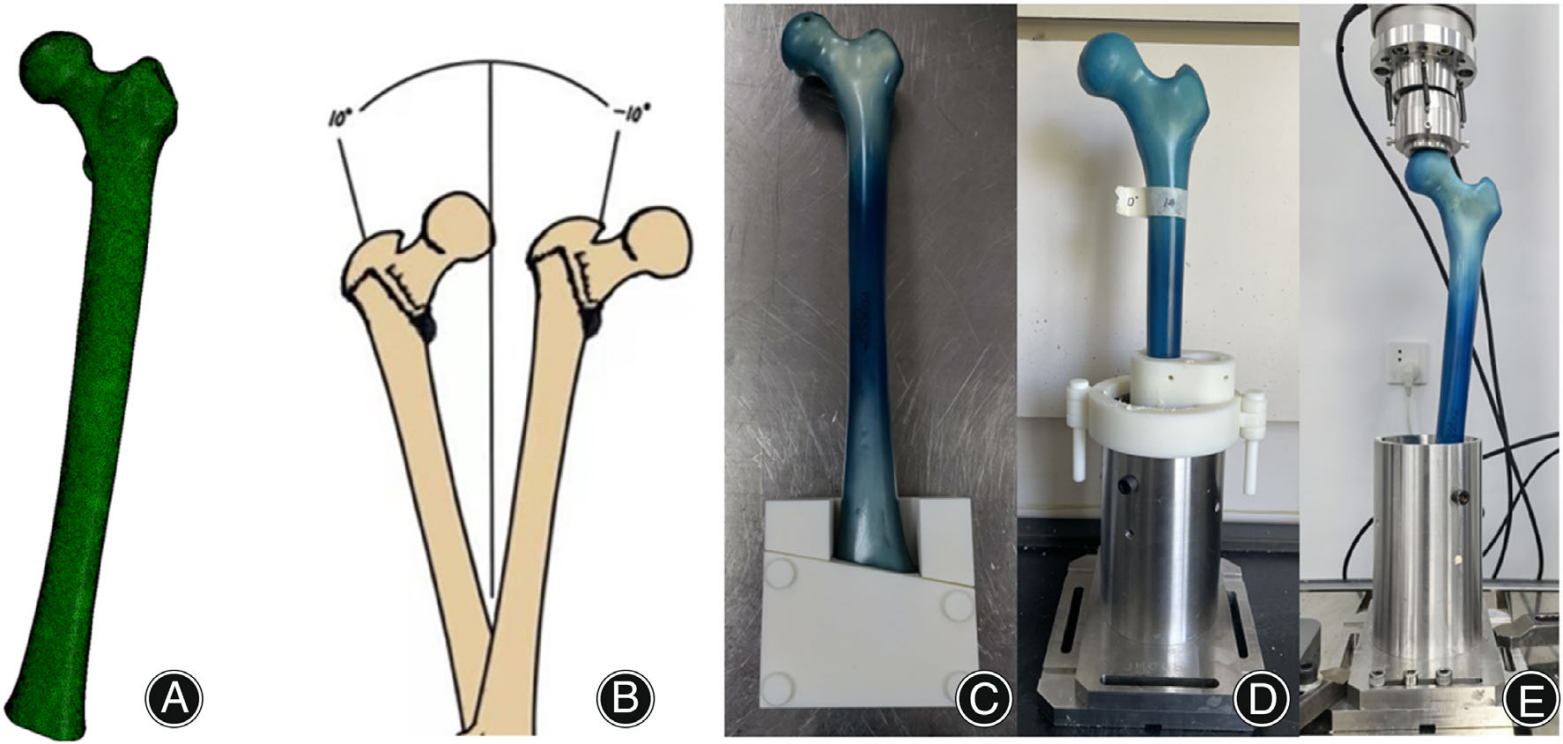
The truncated digital femur was processed by meshing (A) for element creation. five different *α* angles ranging from 10° to −10° (B) were selected for embedding of the 3D femoral model in the FEA and the artificial femur used in the biomechanical experiment. The tooling for osteotomy (C) and embedding (D) of the artificial femur were designed and assembled. The embedded sawbone was assembled on to the biomechanical testing machine (E).

A 73 years old female subject was recruited in our study, and the HRCT of her left lower extremity with the resolution of 2 ppi (adequate for modulation of the structure of bone trabeculae) was acquired and conducted into Mimics20 (Materialise, Leuven, Belgium) for gray scale (Hu) stratification (20 layers), resulting in successful construction of the three‐dimensional femoral geometric model (parasolid (.X_T) file). Then, the 3D model was cut using geomagic design X (3D systems, Rock Hill, SC, USA). After 5.5 cm of the distal end was removed, the femoral model was directly conducted into 3‐matic research 12.0 (Materialise, Leuven, Belgium) for mesh processing. The numbers of unit and node were set as 196,194 and 288,879, respectively, resulting in the construction of element (C3D10) with the size of 3.5 mm (Fig. 2A). Afterward, each layer of stratified gray scales of the digital model file was assigned with the values of density (*ρ*), elastic modulus (*E*) and Poisson's ratio (*μ*) (*ρ* = 0.04 + 0.0008 Hu; *E* = 10200*ρ* ^2.01^; *μ* = 0.3) to create a corresponding material property. The digital simulation process was finally accomplished as the geometric model was embedded, applied with load and boundary condition, solved and verified of convergence in abaqus 2017 (Dassault Systems, Vélizy‐Villacoublay, France). To be specific, the model was embedded for 10 cm in five different *α* angles (10°, 5°, 0°, −5°, −10°, Fig. 2B). For each set of the coronal plane angles, a circular area (radius as 15 mm) with femoral head as the center was projected on to the upper surface of femoral head. As the reference point, the center of the femoral head was applied with a vertical downward load of 3BW (2100N) after its coupling with the projected area.^2^ Under the same embedding and loading condition, the difference in maximum stress between the models mashed with the elements of 3, 3.5 and 4 mm was less than 5%, which fulfilled the requirement for convergence. The equivalent stress distribution of femur were calculated by abaqus 2017 (Dassault Systems, Vélizy‐Villacoublay, France). To rule out the influence of constraint of distal femur on the precise acquisition of stress distribution at the segment of femoral shaft near embedment, an extra 10 mm of shaft was exposed with the corresponding result deleted in the final data.

### Osteotomy and Embedding of Specimen

Fifteen fourth‐generation osteoporosic composite artificial left femurs (#3503; Pacific Research Laboratories, Vashon, DC, USA) were purchased in advance.^21^ Referring to the coronal plane angles for embedment selected in the FEA, these artificial femurs were assigned into five groups randomly with three in each group. To ensure the consistency of the osteotomy distance and the embedding angle within each group, the purchased digital model of the fourth‐generation osteoporotic composite artificial femur (#3503; Pacific Research Laboratory; Vashon, DC, USA) was conducted into Unigraphics NX (Siemens, Plano, TX, USA) to design the tooling for osteotomy and embedding. Under the assistance of tooling, the sawbones were truncated and fixed in a cylindrical base using epoxy resin (Fig. 2C,D). The osteotomy distance and the embedding depth were kept in consistence with those in the FEA.

### Biomechanical Experiment

The biomechanical testing machine ElectroaPuls E10000 (INSTRON, Norwood, MA, USA) was used to perform fatigue test on the truncated and embedded sawbones under vertical load (Fig. 2E). In the fatigue test, the starting load was defined as 1.4BW of the subject recruited in the FEA (1050N), and the force ratio and cyclic rate were set as 0.1 and 2 Hz, respectively. The applied load was increased by 10% every 36,000 cycles (5 h) until failure of the artificial femurs,^22^ and the failure cycle and fracture site were recorded. For each set of *α* angles, the experiments were repeated three times to obtain a more accurate result.

### Statistical Analysis

The data analysis work was accomplished by GraphPad Prism version 9 (GraphPad Software, San Diego, CA, USA). The environmental conditions of all biomechanics experiments are completely the same. Considering that more than two groups were divided, mechanical parameters in different coronal plane *α* angles were compared using one‐way ANOVA (due to the limitation of funding, only three samples were included in each group for statistical analysis). Each mechanical parameter was expressed as the mean ± standard deviation, and the significance level is set at *P* < 0.05.

## Results

### Equivalent Stress Distribution

**Fig. 3:**
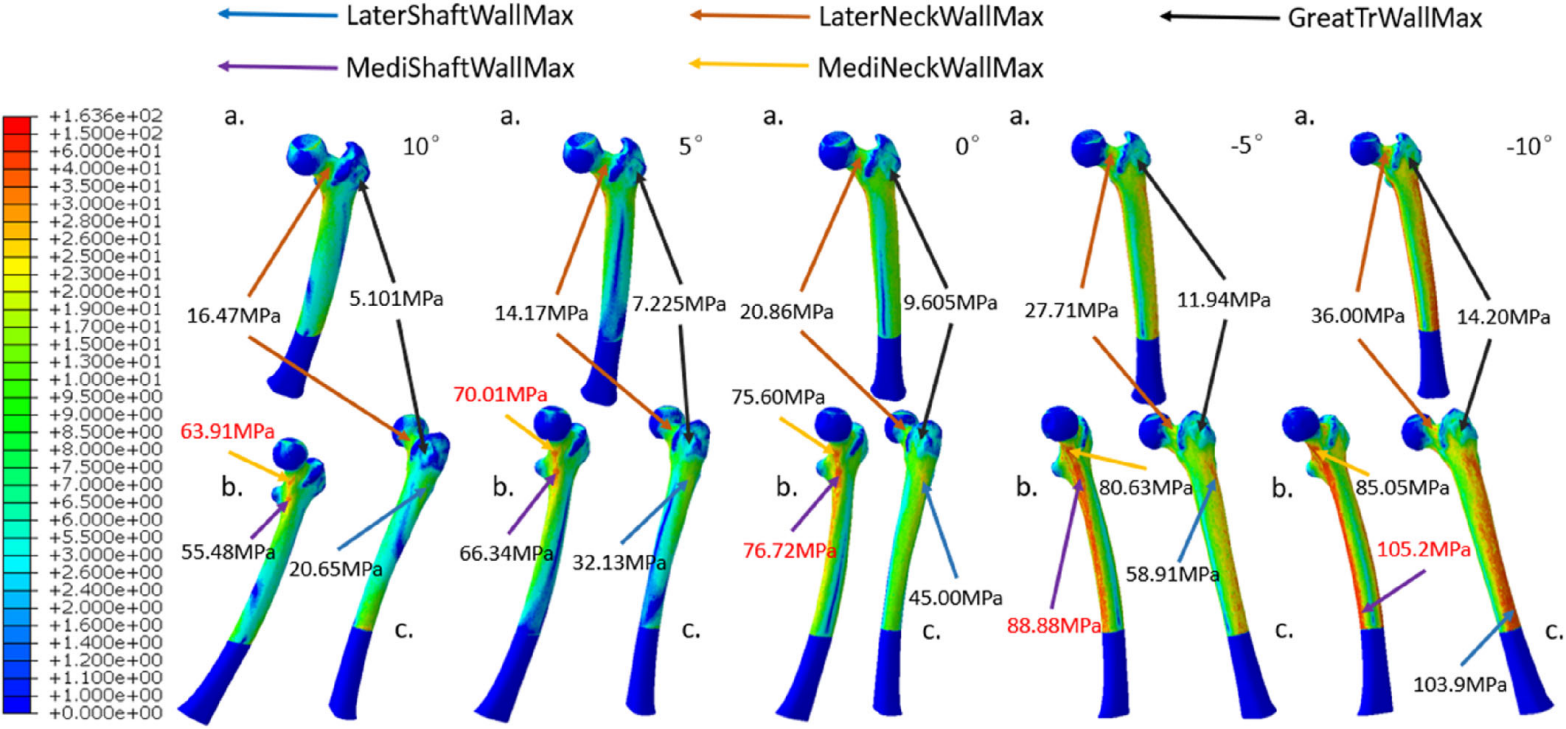
With the *α* angles of 10°, 5°, 0°, −5° and − 10° (from left to right), the equivalent stress distributions of the whole femur under a vertical load of 2100N were acquired. For each configuration, the coronal (A), medial (B), and lateral views (C) of the femur were displayed for the purpose of illustration. The local maximum stress at lateral femoral shaft wall (LaterShaftWallMax), medial femoral shaft wall (MediShaftWallMax), lateral femoral neck wall (LaterNeckWallMax), medial femoral neck wall (MediNeckWallMax), and femoral greater trochanteric wall (GreatTrWallMax) were specified as shown.

According to the result of equivalent stress distribution of the entire femur (Fig. 3), the stress was mainly distributed at the medial wall of the femoral neck and the superior boarder of the medial femoral shaft cortex under physiological configuration (*α* = 10°). The maximum stress of the femur was located below the femoral neck, which was only 63.91 MPa. As the *α* angle decreased, the stress concentration point of the whole femur gradually shifted downward from the medial wall of the femoral neck to the medial and lateral walls of the lower femoral shaft. As the *α* angle was −10°, the maximum stress of the femur occurred at the lower segment of medial femoral shaft cortex with a value of 105.2 MPa.

**Fig. 4:**
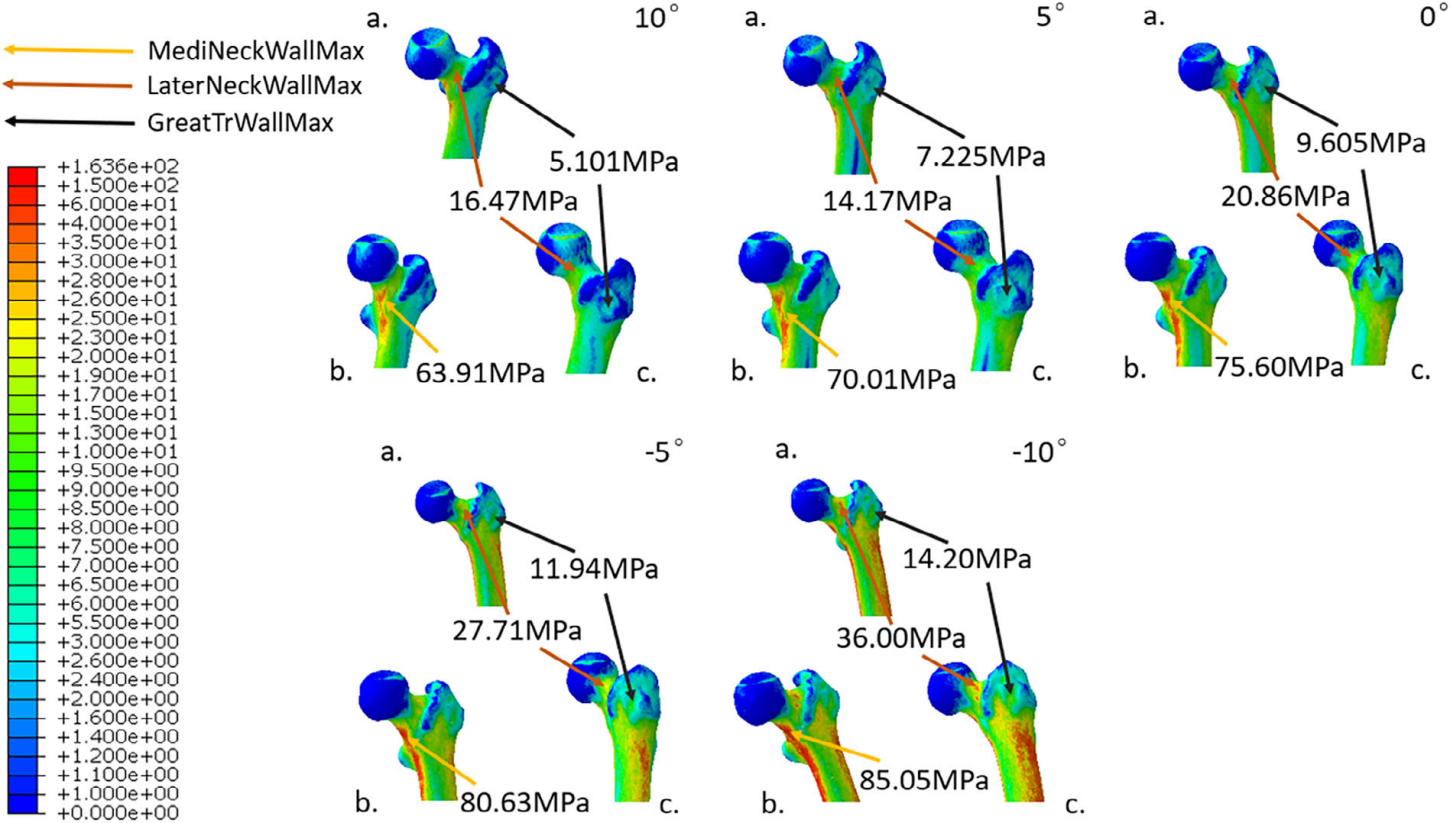
Under digital simulation by the FEA, the equivalent stress distributions of the local proximal femur with the five selected *α* angles ranged from 10° to −10° were calculated. Letter labels were used to demostrate the coronal (A), medial (B), and lateral (C) perspectives of proximal femur. The strategies applied to represent local maximum stress for proximal femur were the same as those used for the femur as a whole in Fig. 3.

**Fig. 5:**
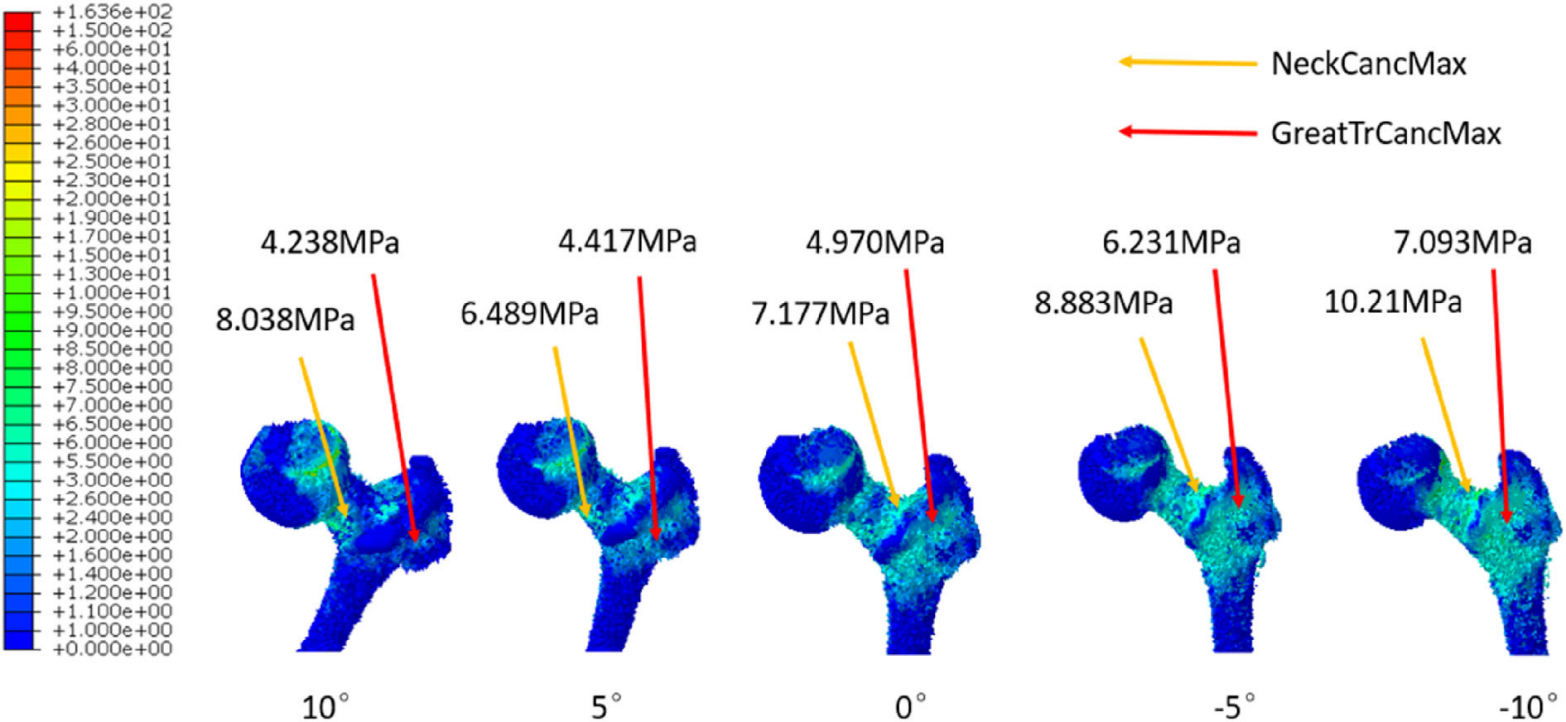
The equivalent stress distributions of local proximal femoral cancellous bone with 10°, 5°, 0°, −5° and −10° (from left to right) as the values of *α* angle were obtained. The methods utilized to indicate local maximum stress for proximal femoral cancellous bone were identical to those used for the entire femur in Fig. 3.

With increasing extent of hip abduction, the stresses distributed in the regions within the proximal femur including the medial femoral neck cortex, the lateral femoral neck cortex, and the femoral greater trochanteric cortex were all increased (Fig. 4). Among them, the greater trochanteric wall showed the largest stress increment. Under extreme hip abduction (*α* = −10°), the corresponding maximum stress increased by 2.78 times compared to that in physiological configuration. On the other hand, the maximum stresses of medial and lateral femoral neck walls increased by 1.33 and 2.19 times, respectively. Similarly, the increment in maximum stress of femoral greater trochanteric cancellous bone (1.67 times) was higher than that of femoral neck cancellous bone (1.27 times) (Fig. 5).

### Failure Cycle

**Fig. 6:**
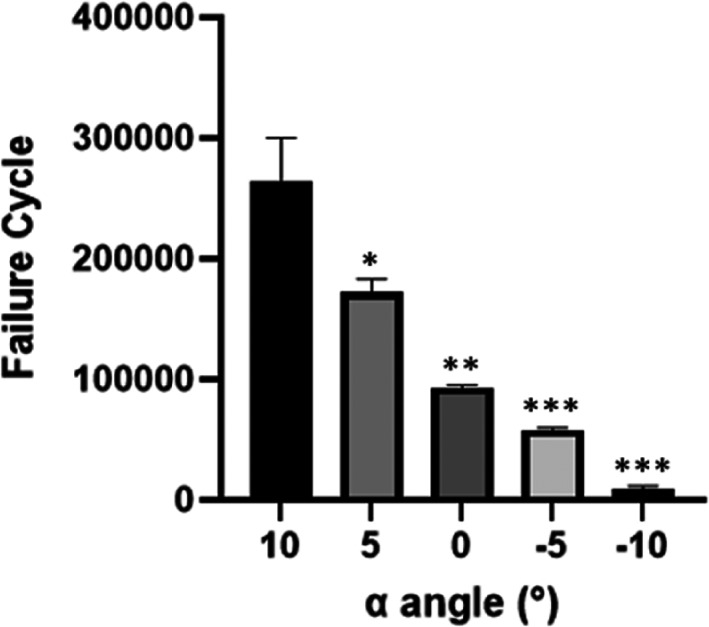
the failure cycles of the artificial femur with the *α* angles limited between 10° and −10° with a declining ladder of 5°. (*P < 0.05).

As shown by Fig. 6, the synthetic bone required a smaller number of cycles to achieve failure when fixed in more abducted configuration, and the statistical significance of the decrement in average failure cycle of the femurs with lower *α* angle compared with those in physiological state showed increasing levels of confidence.

### Fracture Site

**Fig. 7:**
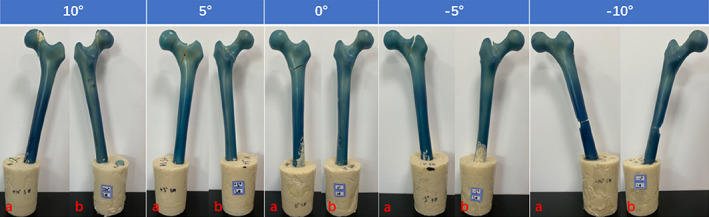
The most representative fracture sites appeared on the sawbones exhibited in front (A) and back (B) in each *α* angle group.

In the fatigue test, the failure sites of the sawbones with physiological coronal angle withstanding reciprocating axial compression were all located at the femoral neck subcephalicly. As the angle formed by femoral shaft and vertical axis decreased, the fracture line has a clear tendency of moving toward the distal end of femur. While the breakage was mostly found at the femoral neck base for the artificial femurs in the 5° group, 2 of the 3 samples in the 0° group had their fracture lines located at subtrochanteric area. When the *α* angle was −5°, all traumas on the femoral models were categorized as either intertrochanteric or subtrochanteric fracture. During extreme hip abduction (*α* = −10°), failures at femoral shaft were witnessed (Fig. 7).

## Discussion

### Elevated Hip Fracture Occurrence with Increasing Hip Abduction

According to the results of our study, the coronal configuration of the proximal femur indeed has significant impact on its mechanical properties. As the coronal configuration gradually changed from physiological state (*α* = 10°) to extreme abduction (*α* = −10°), a general increment in the strain of the proximal femur was observed from equivalent stress distribution of the FEA, which indicates an exacerbation in deformation of the proximal femur with increasing hip abduction. The increased deformation of the proximal femur suggests higher possibility of fatigue injury accumulation during regular ambulation. This hip abduction induced enhancement of fatigue damage accumulation is confirmed by the fatigue test result: the number of cycles required for the failure of the artificial femoral model decreased with the decrement of the *α* angle. Researchers have pointed out that long‐term fatigue damage will lead to significant reductions in the strength and stiffness of canine proximal femur,^23^ believing that the microcrack caused by the fatigue damage would make the proximal femur more fragile and prone to fracture. Similarly, it is reasonable to speculate that the augmented accumulation of fatigue injury on the proximal femur might also lead to relatively higher risk of local microcrack under extreme abduction configuration and elevated incidence of hip fracture during sideway fall.

The coronal configurations of proximal femur designed in this study are not the “special cases” that rarely happen in real life. In fact, serious malalignment of lower body force line is very common in patients with degenerative knee disease.^24^ Among the clinical cases of arthritic knees, the number of genu varum is much higher than that of genu valgum (73.8% vs. 21.1%).^25^ Since genu varum would lead to compensatory hip abduction,^17^ we can rationally infer that the patient combined with genu varum deformity possesses relatively higher risk of hip fracture. In this way, the knee‐targeted surgical intervention that correct hip abduction may exert additional effect of hip fracture prevention.

### Higher Fraction of Trochanteric Fracture Subtype in More Abducted Proximal Femur

In this study, the location of femoral breakage under a variety of coronal configurations is also within our scope of interest. According to the stress distribution result, the maximum stress of the whole femur was situated below the femoral neck under physiological angle, which endows the maximum deformation and fatigue injury accumulation at the identical area. Thus, the fracture line occurred at the femoral neck observed in the fatigue test is explained. Although the stress concentration point of the femur shows the tendency of shifting toward femoral shaft with decreasing *α* angle, the relatively higher strength of femoral shaft compared to that of proximal femur prevent the fatigue injury accumulated at the shaft from causing its failure. Hence, no fracture line occurred at femoral shaft was detected, except in the −10° group. On the other hand, the stress increment of the local proximal femur during hip abduction is most prominent at greater trochanteric area. Although the neck region constantly holds the maximum stress locally, the relative lower bone density and strength of the femoral greater trochanter compared with those of thefemoral neck make this part prone to fatigue injury accumulation especially when the proximal femur is in the more abducted configurations,^26^ which is consistent with the more frequent appearance of a fracture line near the trochanteric area under the *α* angles of 0 and −5°. If the compensatory hip abduction caused by genu varum could lead to the increase of microcracks and corresponding decrease of strength and stiffness at the greater trochanteric region under axial compression, we shall further verify the correlation between lower body force line and hip fracture type (femoral neck fracture *vs* trochanteric fracture) via clinical experimental approach.

### Validation of “Fulcrum‐Balance‐Reconstruction” Model

**Fig. 8:**
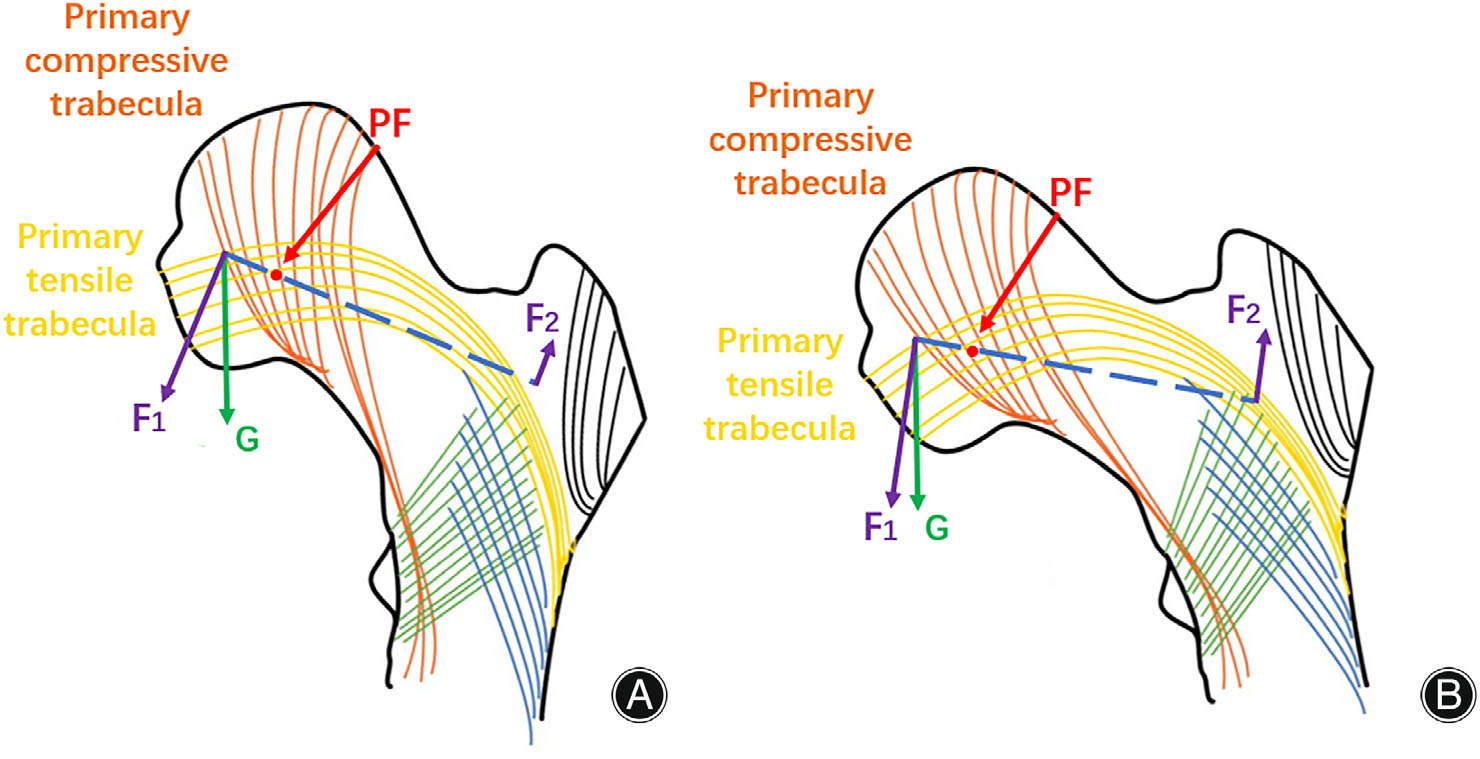
The mechanical analysis of proximal femur in physiological configuration (A) and extreme abducted configuration (B) using “fulcrum‐balance‐reconstruction” model. G, the gravitational force of body weight; F_1_, the division of body gravity perpendicular to the physiological lever; F_2_, the tensile stress generated within greater trochanter during the weight exertion at femoral head; PF, physiological fulcrum.

As the degree of hip abduction exacerbated, a marked increment in the stress distributed at greater trochanter occurred. This result serves as a validation for the theoretical model “fulcrum‐balance‐reconstruction” we proposed based on our previous clinical experiences and knowledge of the structure of proximal femur.^27^, ^28^ According to our hypothesis, the proximal femur contains a special laborious lever structure with the physiological fulcrum (PF) located at the overlapping zone of tension trabeculae and compression trabeculae (Fig. 8). When the gravity from upper body is exerted on the femoral head, the force component (F1) perpendicular to the lever tries to move the lever with a very short moment arm. In this case, the tensile stress generated within the tension trabeculae at the greater trochanteric region (F2) would oppose the moment formed by gravity with a rather long moment arm. Therefore, the stress within the greater trochanter is much smaller than the body gravity. Under physiological conditions, this lever has extremely high stability and excellent mechanical property. As the coronal configuration of proximal femur is increasingly abducted, F1 would increase accordingly. Since the lengths of the two moment arms at both ends of the physiological fulcrum are constant, F2 would also increase correspondingly. This prediction is consistent with the experimental result of the stress distribution for total proximal femur and proximal femoral cancellous bone (Figs 4 and 5).

### Strengths and Limitations

Certainly, there are some shortcomings in our research. First of all, this is a pure experimental study that lacks sufficient clinical evidence. Moreover, it needs a more accurate mathematical model to describe the fulcrum site and the stress distribution. Even though Sawbones is a widely used artificial model that is believed to mimic human bone structure to a certain extent, it still cannot completely replace the role of cadaver bone in biomechanical research. Furthermore, the variance of the fracture site observed in the same angle group reflected that minor inconsistency existed between the specimens. Further research carried out with cadaver bone or synthetic femur with higher consistency is expected in the future. Considering the change of the muscle and ligament attached to the femoral trochanter during hip abduction, we believe that the influence exerted by soft tissue on the mechanical property of the proximal femur can potentially be a new direction of research in the subsequent studies. By carrying out related clinical research, we could not only investigate the correlation between lower body force line and hip fracture incidence/subtype of hip fracture but also further verify and correct our “fulcrum‐balance‐reconstruction” hypothesis. Although this model is a relatively simple concept, we believe that it can bring researchers with the inspiration of new treatments for proximal femoral fractures and innovative designs of internal fixation devices.

### Conclusion

Overall, more abducted femoral coronal configuration has detrimental impact on the mechanical properties of proximal femur. The increased strain at local proximal femur and reduced fracture cycle for the synthetic femoral model with increasing hip abduction indicated higher incidence of hip fracture, while the relatively higher increment of stress at greater trochanter region and the migration of fracture line away from the proximal femur during hip abduction suggested larger fraction of trochanteric fracture in the proximal femur with more abducted configuration. In addition, our theoretical model “fulcrum‐balance‐reconstruction” was supported by the obvious augmentation of the stress distributed at femoral greater trochanter.

## Author Contributions

Lijia Zhang: designed the finite element analysis and biomechanical experiments; analyzed the data; composed and revised the manuscript. Baozhang Zhu: designed the biomechanical experiments. Liwan Chen: performed the finite element analysis. Wenqing Wang: performed the biomechanical experiments. Xiaoyong Zhang: designed the tooling for osteotomy and embedding. Jianguo Zhang: supervised the study.

## Ethics Statement

The study was approved by the institutional ethics committee of Peking Union Medical College Hospital, Beijing (IRB/IEC No: K22C0563).
